# What is effective communication in breastfeeding care? Perspectives from Latina women

**DOI:** 10.1371/journal.pone.0325592

**Published:** 2025-06-26

**Authors:** Deanna Nardella, Sofia I. Morales, Rafael Pérez-Escamilla, Genesis Vicente, Leslie Brown, Natasha Ray, Kathleen O’Connor Duffany, Elizabeth C. Rhodes

**Affiliations:** 1 Department of Pediatrics, Yale School of Medicine, New Haven, Connecticut, United States of America; 2 Department of Social and Behavioral Sciences, Yale School of Public Health, New Haven, Connecticut, United States of America; 3 Yale Griffin Prevention Research Center, New Haven, Connecticut, United States of America; 4 Community Alliance for Research and Engagement (CARE), New Haven, Connecticut, United States of America; 5 Department of Psychiatry, NYU Grossman School of Medicine, New York, New York, United States of America; 6 New Haven Healthy Start, Community Foundation for Greater New Haven, New Haven, Connecticut, United States of America; 7 Hubert Department of Global Health, Rollins School of Public Health, Emory University, Atlanta, Georgia, United States of America; Deakin University Faculty of Health, AUSTRALIA

## Abstract

**Background:**

Despite effective communication being critical to providing person-centered care, little is known of effective communication in breastfeeding care from the perspective of Latina women in the United States (U.S.) who experience breastfeeding inequities. Our study identifies (a) what constitutes effective communication and (b) which provider practices promote or hinder effective communication in the context of breastfeeding care delivered within the pregnancy, delivery, and postpartum periods to Latina women in the U.S.

**Methods:**

We analyzed data from a community-engaged study that included semi-structured interviews in English or Spanish with Latina women from low-income households in Connecticut. Women were asked about communication experiences with healthcare providers during their breastfeeding care across the pregnancy, delivery, and postpartum periods. Reflexive thematic analysis was used to analyze the data and develop a framework depicting key themes.

**Results:**

Of the 21 women interviewed, approximately half were 25–31 years of age (48%), were born outside of the U.S. (52%), and most had prior children (86%). We identified two themes of what constitutes effective communication in breastfeeding care to Latina women: personalized breadth and depth of breastfeeding information (theme 1) and bidirectional exchange of information with providers (theme 2). Provider use of open-ended questions that explored women’s breastfeeding experiences, goals, and challenges asked across the breastfeeding care continuum promoted effective communication. Conversely, providers who asked about breastfeeding at a single visit and/or used rushed, checklist-style questioning left women with unmet information needs and hindered effective communication. While some women preferred communication aligned with their cultural and language preferences, others appreciated providers who engaged with non-fluent Spanish, seen as a supportive gesture. Our *“Framework for Effective Communication in Breastfeeding Care”* illustrates our findings.

**Conclusion:**

Our findings could inform provider and systems level efforts to promote more effective communication in breastfeeding care, ultimately enhancing care quality and person-centeredness.

## Introduction

To deliver high-quality healthcare, it is critical that it be person-centered— or care that is respectful of and responsive to individual preferences, needs, and values and ensures that people’s values guide all clinical decisions [1–6]. Women want person-centered maternity care [2,4]; effective communication is key to providing person-centered maternity care [1–5,7]. Communication factors explain over 70% of the variation in patients’ satisfaction with maternity care [8]. Promoting more effective communication in maternity care [3], and in particular breastfeeding care [8–13], is likely to facilitate a more balanced exchange of information, values, and expectations between patients and providers, in turn improving patient trust, autonomy, and satisfaction with care, as well as health outcomes [2–4,11,14].

Communication on infant feeding, including breastfeeding, is a high-priority, emotionally laden discussion for many women, seen to be tied to one’s sense of motherhood [4,15–20]. Women across global regions and socioeconomic strata have reported poor communication experiences with providers around breastfeeding [8–13], recommending that healthcare providers who support breastfeeding women obtain dedicated training in communication skills [21]. Despite acknowledging their important role in infant feeding decisions, maternal-infant healthcare providers feel under prepared, fearful of eliciting guilt, or left with insufficient time to provide adequate breastfeeding education and support to families [12,22–27]. Moreover, previous studies have identified a gap between what women and providers consider optimal breastfeeding support, including breastfeeding counseling [12,26], with most women reporting negative or no breastfeeding support from their providers [28].

Promoting effective communication in breastfeeding care is not only key for delivering person-centered, high-quality care for individuals, but has potential to mitigate persistent inequities in breastfeeding outcomes for Hispanic women and children in the United States (U.S.) [29]. Women with Hispanic ethnicity and Spanish as a primary language have lower breastfeeding exclusivity and duration compared with non-Hispanic populations [30,31]. Nearly half (45%) of U.S. women report refraining from voicing concerns with their maternity providers [32] and Spanish-speaking women, specifically, report being unsure of who to ask for breastfeeding educational support [33]. Communication breakdowns such as these are likely to negatively impact breastfeeding outcomes and contribute to breastfeeding inequities, as maternal comprehension of breastfeeding information from providers has been directly correlated with exclusive breastfeeding during birth hospitalization [34].

Most prior studies exploring women’s experiences of breastfeeding care were not conducted in the U.S. [11,35–41] or solely captured the voices of U.S. women of non-Hispanic Black and White race and ethnicity [11–13,35,36,42]. Of U.S. studies that included Hispanic women, Hispanic participants constituted a minority of the sample, were mostly English-speaking, and their experiences of effective communication with healthcare providers in the context of breastfeeding care across the maternal and infant care continuum were not specifically explored [13,28,42–49]. As such, U.S. English and Spanish speaking Hispanic and Latina women’s experiences communicating with providers during their breastfeeding care remains poorly understood.

We aimed to identify what constitutes effective communication in breastfeeding care and which provider practices promote or hinder effective communication in breastfeeding care delivered across the pregnancy, delivery, and postpartum care continuum from the perspective of English and Spanish-speaking Latina women in the U.S. Here, we define breastfeeding care as all breastfeeding education and support delivered within a healthcare setting across the breastfeeding journey encompassing the pregnancy, delivery and postpartum periods. Our findings elevate the voices of Latina women and can inform efforts at the individual and systems levels to promote more effective communication between women and their providers, a necessary step to delivering high-quality, person-centered breastfeeding care and advancing health equity in breastfeeding care, experiences, and outcomes.

## Methods

We conducted a secondary analysis of data collected as part of a larger community-engaged qualitative study to describe the experiences of breastfeeding care for Hispanic and Latina women from the Greater New Haven area in Connecticut (U.S.). This larger study was conducted in partnership with The New Haven Breastfeeding Task Force and New Haven Healthy Start, local community organizations who serve mothers and children. Our team consisted of masters and doctoral-level public health researchers and program managers from community-engaged research centers (Community Alliance for Research and Engagement (CARE) and Yale-Griffin Prevention Research Center; SIM, RPE, GV, KOD, ECR), a practicing pediatrician, physician-scientist, and lactation consultant (DN), a community leader from Healthy Start (NR), and a community research fellow (a community member with lived experience who participated in training on research principles and methodologies and meaningfully contributed to the research process; LB). Most team members identify as Hispanic and Latina.

Acknowledging the potential impact of our subjectivity on the research process and the importance of practicing personal and interpersonal reflexivity, we routinely discussed our positionality and how our personal lived and professional experiences could influence data collection and analysis. During data collection, we were mindful to develop interview guides and probes that were neutral and intended to elicit both positive and negative experiences of breastfeeding care within each care period. We also held regular discussions about the data throughout data analysis, practicing reflexivity while also making sure that the findings were firmly grounded in the data.

### Study sample and data collection

The study included women who identified as Hispanic or Latina heritage, were 18 years or older, lived within a low-income household, had a prenatal intention to breastfeed, no medical contraindication to breastfeeding, and received prenatal, delivery, and postnatal medical care in the Greater New Haven area. Low-income household was determined by enrollment in Medicaid or Supplemental Nutrition Assistance Program (SNAP) through Women, Infants and Children (WIC), or eligibility for SNAP as determined by self-reported household income. Women were recruited by purposive and snowball sampling. Our team engaged in extensive in-person recruitment at community events, such as food drives and health fairs. Additionally, we actively promoted the study through social media, including local Facebook groups for mothers. Community and governmental organizations, including New Haven Healthy Start and WIC, among others, supported recruitment efforts by sharing the study flyer through social media and distributing physical flyers. Women who participated in an interview were also encouraged to inform their family members and friends about the project.

We completed semi-structured interviews via video or phone in participants’ preferred language (English or Spanish) between October 28, 2021 and December 27, 2021. The study team developed a semi-structured interview guide to elicit women’s experiences of breastfeeding care within the pregnancy, delivery, and postpartum periods, as well as across the three experience of care domains identified in the World Health Organization (WHO) Quality of Care Framework for Maternal and Newborn Health [7]. Women were asked to describe their experiences and perceptions of breastfeeding care received within each care period and what they would have wanted for themselves and Latina women more broadly. The interview guide was developed in English, then translated into Spanish by a community research center, CARE, and once more reviewed for accuracy by author and native Spanish speaker, SIM. Both guides were pilot tested and refined together with our community partners. Question stems and example probes used to guide each interview can be found in our S1 Table.

Each participant was interviewed at a single timepoint for 60–90 minutes. Our interviewers, authors SIM and GV, both identify as Latina women and are bilingual in English and Spanish. Both interviewers took field notes during each interview. As part of an iterative data collection process, post-interview team debriefs were held to review field notes, identify topics raised by participants during interviews, and add questions to the guides to delve deeper into these topics in subsequent interviews. These debriefs were also used to discuss saturation, with a dual focus on what topics were identified (code saturation) and the extent to which these topics were well understood (meaning saturation) [50]. After 21 interviews, the team determined through a group consensus process that saturation had been reached and stopped data collection [51]. All interviews were audio-recorded, transcribed, and translated into English by a professional transcription/translation company. Authors de-identified and reviewed each transcription to ensure completeness and accuracy. A thank you letter, $45 gift card, and a community resources packet were sent by mail to each participant once the interview was complete.

### Data analysis

As part of the larger qualitative study, the textual data were coded in MAXQDA 2020 using codes that were organized according to the three experience of care domains put forth by the WHO Quality of Care Framework for Maternal and Newborn Care: effective communication, respect and dignity, and emotional support. Rich detail was found around women’s experiences of effective communication with providers. As such, first author DN led a secondary analysis of textual data that had been coded with the subset of codes related to the effective communication domain, using reflexive thematic analysis [52,53]. The core analytic team included DN and co-authors who had collected the data, read all transcripts, and coded the data as part of the larger qualitative study. An analysis into the other experience of care domains (as outlined within the WHO Quality of Care Framework for Maternal and Newborn Health [7]) is ongoing and will be presented in a separate manuscript.

For the secondary analysis, the core analytic team became further immersed and intimately familiar with the data related to the effective communication domain, reading the textual data multiple times and taking notes that captured initial analytic observations and insights. We then coded the data and developed potential themes by examining the codes and coded text segments. Next, we checked the potential themes against the coded text segments and further developed the themes, which involved combining related sub-themes. We then named, wrote, and refined thick descriptions of each theme. Finally, we developed a framework to depict these themes.

### Terminology and ethical considerations

Our team recognizes the importance of using inclusive language when discussing infant feeding techniques used among all birthing persons [54–57]. We understand that individuals identify with different ethnic groups and titles, including Hispanic, Latina, Latinx, and Latine, among others. As part of our community-engaged approach, thoughtful discussions were had with our partners around appropriate terminology for our study. Together, the decision was made to use terms such as “woman,” “women,” “breastfeeding,” and “Latina” in our recruitment process to best represent the population of focus for this specific study. These terms are used in this manuscript to accurately describe the individuals who were recruited for and participated in this study.

The term “provider” in this manuscript represents all individuals who may care for women and infants within the U.S. medical system. The phrase “care team” refers to the collective team of providers caring for women and infants across the pregnancy, delivery, and postpartum periods. Provider types discussed by women in our study included nurses, midwives, advanced practice practitioners, physicians, and lactation consultants. Here in, the phrase “maternal and infant care continuum” refers to the pregnancy, delivery, and postpartum care periods.

Our study was determined to be exempt from annual review by the Yale University Institutional Review Board (IRB) under 45CFR46.104 [2](ii). Nonetheless, informed consent was obtained from each participant before the interview.

## Results

**Fig 1:**
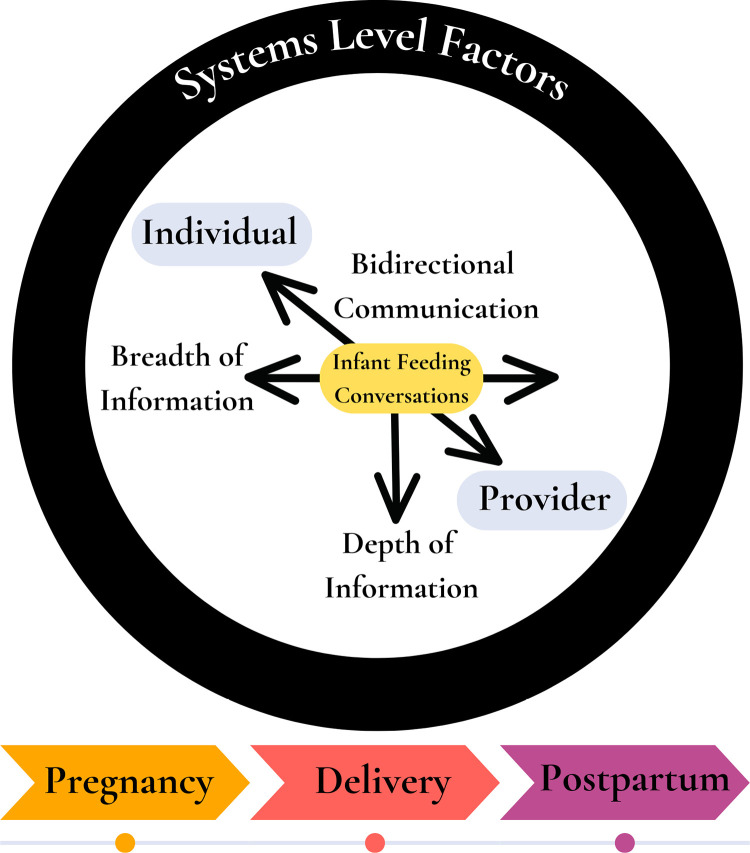
Framework for effective communication in breastfeeding care. Fig 1 illustrates the two key themes of effective communication, with arrows depicting the importance of breadth, depth and bidirectional exchange of breastfeeding information between women and their providers. The figure also highlights the presence of systems level factors that influence effective communication in breastfeeding care, as well as women’s belief that breastfeeding conversations delivered across the maternal and infant care continuum promote effective communication.

Of the 21 Latina women included in our sample, about half of women were 25–31 years of age (48%) and born outside of the U.S. (52%), and most had either a high school education (33%) or an associate or bachelor’s degree (43%). All women with a prior child (86%) had breastfed their prior child(ren) (Table 1). We identified two themes of what constitutes effective communication in breastfeeding care from the perspectives of Latina women: receiving personalized breadth and depth of information from providers that meets their individual needs (theme 1) and engaging in a bidirectional exchange of information with providers (theme 2). We also identified provider practices that either promoted or hindered effective communication during breastfeeding care from women’s perspectives (Table 2). Our *Framework for Effective Communication in Breastfeeding Care* (Fig 1) depicts our themes and highlights systems level factors that influence effective communication between women and their providers. Our framework also highlights women’s belief that effective communication was promoted by having breastfeeding conversations across the maternal and infant care continuum as opposed to a single timepoint in care.

**Table 1:** Description of study participants, all of whom identify as latina women.

Participant Characteristics	N (%)
**Age (years)**	
18-24	4 (19.0)
25-31	10 (47.6)
32-37	5 (23.8)
38-42	2 (9.5)
**Education Level**	
Less than high school	3 (14.3)
Some high school	1 (4.8)
High school graduate or GED	7 (33.3)
Associate’s or Bachelor’s Degree	9 (42.9)
Graduate level or higher	1 (4.8)
**Place of Birth**	
In the U.S.	10 (47.6)
Outside the U.S.	11 (52.4)
**Experience Breastfeeding Previous Infant**	18 (85.7)

**Table 2:** Key themes of effective communication in Breastfeeding (BF)^*^ care.

	Provider Communication Practices	
Communication Theme	Promote Effective Communication	Hinder Effective Communication
***Breadth & Depth of Information***	Personalize conversation to women’s informational needs based on: BF goals and prior experience BF concerns and anticipated barriers Access to and use of pumps Work plans Supports (e.g., partners, peers, family) Cultural influences around BF Explore BF at each clinical encounter across pregnancy, delivery, and postpartum Provide anticipatory guidance on potential BF challenges and how to address them	Do not explore and/or personalize BF information to meet women’s individual needs Discuss BF at a single clinical encounter or not at all Provide inaccurate or inconsistent BF information between providers Do not consider women’s culture(s) & language(s) when providing verbal or written BF information, assume women understand information provided in English
***Bidirectional Communication***	Use open-ended questions around BF Ask about women’s BF goals Create opportunities for women to share BF questions or concerns early on to guide conversation and meet women’s needs across the care continuum Ensure women’s understanding and comprehension of BF information and BF care plan Explain how women’s BF goals were considered, and ideally supported, in the medical plan of mother and infant	Use close-ended, “checklist” style questions around BF Assume or do not ask about women’s BF goals Do not ask if women have BF questions or concerns Do not ensure that women understand BF information and BF care plan Do not explain how women’s BF goals were considered, and ideally supported, in the medical plan of mother and infant (particularly around formula supplementation) Provide pamphlets or packets without verbal explanation of their content

* BF used as an abbreviation for breastfeeding.

### 1. Personalized breadth and depth of breastfeeding information

#### 1a. Sufficient breadth and depth of information.

To Latina women, effective communication in breastfeeding care consisted of conversations with providers that were personalized to the breadth and depth of information necessary to meet women’s individual informational needs. Women who experienced breastfeeding conversations that met their informational needs in turn felt supported, prepared to breastfeed, and “powerful.”

Women valued providers whose communication went “beyond the basics” of breastfeeding benefits, using open-ended questions to identify women’s breastfeeding experiences and goals, available social support(s), and anticipated or lived barriers to breastfeeding success. Women liked when providers used open-ended questioning to tailor their communication to meet their individual informational and emotional needs. One mother noted how the use of open-ended, exploratory questions by her pediatric provider made her feel supported as a mother, expecting the focus to only be on her infant:

*She (pediatric provider) asked me if I needed support with breastfeeding, if—how I was doing with it and things like that. Like, she was just asking me where I stood with it and, if I needed any help, that she was willing to provide me with resources... I found it h—very helpful ‘cause, I mean, she’s a pediatrician. I wouldn’t think that they would, you know, pay mind to the mother. [Laughter] I thought it was all about the kids, but, in a way, it is helping the kids if they’re—have a-a mom who’s well-supported and has the help that she needs to be a good mom. [25 years old, with previous breastfeeding experience]*

Women received sufficient breadth and depth of breastfeeding information not only through open-ended questions, but also through ongoing conversations with their providers. Provider communication was seen as most effective when tailored breastfeeding discussions occurred at each clinical encounter throughout the care relationship, as women’s informational needs evolved over time. Providers who routinely discussed breastfeeding made women feel that their breastfeeding knowledge was “confirmed” or “reinforced,” altogether enhancing women’s sense of physical and emotional breastfeeding support from their care team and self-efficacy with breastfeeding. Moreover, several women identified providers revisiting breastfeeding conversations throughout the care relationship as one of the most satisfying aspects of their care. When asked what she was most satisfied with in her breastfeeding care, one woman shared:

*The fact that they (pediatrician) are still questioning me, to this day, abou—like, at her appointments like, “Oh, are you doing okay? Do you need help?” They-they still question me, even at these appointments, to see how she’s doing, breastfeeding wise, if I’m doing good, if I need help—anything like that. [24 years old, with previous breastfeeding experience]*

Providers who routinely engaged women with open-ended questions, going “beyond the basics” to also discuss women’s breastfeeding concerns or challenges, were described by women as non-judgmental, “attentive,” “kind,” “open,” “honest and real,” and overall seen as invested in women’s breastfeeding journeys. Women trusted providers who engaged in this manner, sharing that they were not only comfortable sharing breastfeeding questions and concerns, but intentionally returned to these providers for their family’s care needs.

Women valued when breastfeeding conversations were tailored to their language and cultural preferences as well. For some women, communicating in their preferred verbal and written language or with racially and ethnically concordant providers allowed them to more openly and accurately share their concerns and curiosities about breastfeeding, in turn enabling providers to be more responsive to women’s informational needs. One woman shared:

*They should have Latino staff. Not everyone speaks English. They should have more people who can provide us information about breastfeeding, how important it is, because some people do understand its importance but there are people who don’t. We need information and all that. [40 years old, with previous breastfeeding experience]*

For women who spoke Spanish, specifically, some appreciated when providers discussed breastfeeding with “imperfect” Spanish, seen as an effort to connect and a display of investment in women’s breastfeeding experience. When asked if her provider conversing with non-fluent Spanish impacted the breastfeeding support she received, one woman explained:

*I think that perhaps to a certain point when you might want to discuss in depth some specific topic, where you might think, she’s not going to understand me. However, I also see that they try to make it easy, they look for a translator if you can’t—yes—interact well with them. I also see that they are looking for a way for you to feel comfortable and at ease talking to her, that one can express what one wants. [42 years old, with previous breastfeeding experience]*

Other women preferred speaking with providers and/or translators who were fluent in Spanish for all breastfeeding conversations to ensure comprehension. Many women reported that interpretive services were used across healthcare settings. A few women reported that nursing staff or a family member or partner served as their interpreter , which was viewed as acceptable modes of translation during their breastfeeding care.

Few women also valued when providers considered women’s cultural norms when providing information. One woman shared that she appreciated how the advice she was given on maternal nutrition while breastfeeding included foods that were common within the Hispanic community, in turn making her more likely to follow the recommendations provided.

#### 1b. Insufficient breadth and depth of information.

Ineffective communication experiences were shared when women experienced unmet breastfeeding informational needs in communication with providers, often resulting in negative perceptions of care, as exemplified with one woman describing her breastfeeding care experience as “a little bit bittersweet, because I didn’t feel like at the end I got the information that I needed.” Most women reported discussing breastfeeding with their providers briefly at a single timepoint in the care relationship, feeling as if they were left without opportunities to discuss breastfeeding with their care team as their informational needs evolved over time.

The most extreme instances of unmet informational needs were reported by women whose providers assumed or failed to ask about their breastfeeding plans altogether, resulting in negative perceptions of care and less trust in one’s provider. Highlighting the influence of provider assumptions within breastfeeding conversations, one woman recalled:

*What if I didn’t wanna do it? Or what if I wanted to give half? …She (obstetrician) just said, ‘Oh, I know you’re breastfeeding.’ And that was the first and last time we talked about breastfeeding... She doesn’t know what I went through. She didn’t even ask me ‘how do you feel? I know that you breast fed your first one, how did that go?’ There was no open-ended question with breastfeeding at all... She just assumed. And I didn’t want her to assume. I wanted her to ask me. [29 years old, with previous breastfeeding experience]*

Women’s wanting for enhanced breadth and depth of breastfeeding information from their providers stemmed in part from a retrospective desire for more “realistic” breastfeeding information, or anticipatory guidance on what challenges they may face and how to handle them. Women expected to be more prepared for the challenges of breastfeeding through conversations with their providers, reviewing together “the problems you might go through, [and] if you have a problem, where can you call.” Many women anticipated breastfeeding to come easily, and thus when met with challenges such as low milk supply, pain, or difficulty with infant latch, they shared feeling “frustrated,” “under prepared,” and “uncomfortable.” When asked how she felt about not receiving a lot of information on breastfeeding in pregnancy, one woman shared, “It didn’t make me feel very comfortable. I was feeling like an amateur, out of information, and all that.”

Many women also encountered inaccurate and/or inconsistent breastfeeding information between providers, describing their care team as “not all on the same page” and giving “different information.” Experiencing inaccurate and inconsistent breastfeeding information from different providers spurred confusion and frustration, an emotional stress women considered avoidable had more consistent information been delivered. Describing a nurse who cared for her during her delivery hospitalization, one woman recalled:

*The nurses that I spoke—that I asked questions in the meantime, it seemed like they were not all on the same page. You know? I was gettin’ different information... one would tell me, uh, you have to wake the baby up, uh, and feed him every three hours. The other one would say never wake up the baby. If he’s sleeping at night, don’t wake him up. Uh, the third one came in and was like, naw. You don’t wanna breastfeed him every four hours. You wanna do it every three hours, so it was just—you know, I don’t know, confusing. [30 years old, without previous breastfeeding experience]*

Instances of inconsistent communication that were particularly stress-inducing for women were conversations around the timeframe to achieve a full milk supply, lactation delay after cesarean delivery, and how to build or maintain a milk supply while supplementing with formula. Receiving inaccurate and inconsistent breastfeeding information from providers often resulted in a loss of trust in the care team, as highlighted by one mother, who worked as a breastfeeding peer counselor, describing her experience with a nurse during delivery hospitalization:

*I was actually making a lot. And she (nurse) said, “Oh no. That is-that is not enough. You need to pump more.” I said, “No, that is enough. That is actually more than enough.” She said, “No, no, no. We cannot.” She said, “The baby will not get full with this.” I said, “The baby will get full with it... My son is only a couple of hours old. And colostrum is-it’s about quality not quantity.” She said, “But it won’t even fit in a bottle.”... I was very anxious to come back (from having a chest X-ray) ‘cause I didn’t want her to give him any formula. Even though I told her please do not give him anything till I’m back, but I still didn’t trust her. [29 years old, with previous breastfeeding experience]*

This same mother reported being concerned for other women without her breastfeeding knowledge who would not question if providers’ advice on breastfeeding was accurate, “’Cause you-you see them as experts of the m-of medicine, right? Whatever they tell you is true. You don’t question them.”

Provider communication practices that hindered effective communication by leaving women with unmet informational needs included the use of close-ended or “checklist” style questions, felt to create minimal opportunity for women to guide conversation towards their individual concerns. Merely asking a woman if she was breastfeeding, yes or no, “—it wasn’t helpful. It was just checking on progress, but it wasn’t offering solutions or answers.” With providers who communicated in this manner, women often felt unable—and sometimes uncomfortable—voicing questions and concerns. Some women reported a perception that they were “botherin’ them (providers) with all these questions” on breastfeeding, while others shared a sense of powerlessness in voicing their breastfeeding informational needs to their care team. One woman stated:

*I stay quiet, what can I say? You are not doing your job! No. You go and you know what you know. You would like the doctor to tell you or educate you more, but it doesn’t happen. [35 years old, with previous breastfeeding experience]*

Though most women who spoke Spanish reported interpreter use during conversations with English-speaking providers, few did not. Some women reported that providers made assumptions around women’s understanding of spoken and written English without asking women their preferred language or verbally reviewing written materials provided in English. One woman described:

*They (providers) didn’t speak Spanish. They heard my non-perfect English and they were able to understand me and I understood them, they never asked me whether I wanted an interpreter or anything, no... They determined that I understood, I can manage in English, and that’s why they provided it to me in English but they did not ask me at any point whether I wanted it in Spanish. [35 years old, with previous breastfeeding experience]*

Providers who used ineffective communication techniques in breastfeeding care, including those who (1) engaged women with closed-ended, rushed, and checklist-style questions, (2) provided inaccurate or inconsistent information, (3) did not provide anticipatory guidance on common breastfeeding challenges, or (4) did not tailor conversations to address women’s top concerns, were ultimately perceived as uninvested in women’s breastfeeding experiences and outcomes, just there “to do their job.”

Many women discussed systems level factors that they felt strongly influenced their provider's ability to effectively communicate about breastfeeding. Across the entire maternal and infant care continuum, women reported provider lack of time due to short clinical visits and high clinical workload as hindering providers’ ability to discuss breastfeeding with sufficient detail to meet women’s informational needs. As a result of insufficient time, women perceived breastfeeding conversations as feeling rushed, impersonal, and overall unsatisfactory. Women also remarked on how varied levels of breastfeeding knowledge and expertise between providers led to women receiving inaccurate or inconsistent information about breastfeeding.

### 2. Bidirectional communication between women and providers

#### 2a. Presence of bidirectional communication.

Latina women described positive communication experiences when providers engaged with bidirectional communication. To women, bidirectional communication with providers involved a back-and-forth sharing of information that allowed women to express their infant feeding knowledge and goals, in turn experiencing conversations that better addressed their unique breastfeeding needs and enhancing their trust in providers.

Provider practices that promoted bidirectional communication included: use of open-ended questions to explore women’s breastfeeding knowledge and goals without assumptions; intentionally creating dedicated time and space for women to voice questions or concerns about breastfeeding in conversations; and discussing women’s breastfeeding care plan, including how their breastfeeding goals were considered within, and supported by, the broader maternal or infant medical care plan.

These communication practices facilitated bidirectional conversations with providers that were in accordance with women’s highest concerns related to infant feeding, ensured women’s comprehension of infant feeding information provided by their care team, and allowed for women to feel that providers recognized and were invested in their breastfeeding goals. Appreciating how her midwife provided rich detail on breastfeeding and ensured her understanding with repeated examples, one woman recalled:

*I liked how detailed the midwife was when she was explaining things to me instead of giving me the short, brief way to just say, “Oh this is how you do this. Got it? Good. Okay, bye.” She made sure I understood what was going on, and, you know, went over it again and gave me examples. [23 years old, with previous breastfeeding experience]*

Bidirectional communication was also key to providing culturally and linguistically appropriate breastfeeding care, including use of language-concordant handouts to enhance, and not replace, verbal breastfeeding education. Bidirectional communication was key for eliciting and incorporating women’s cultural beliefs and values into counseling points and providing relevant community-based referrals to follow women’s concerns between clinical encounters, including peer counselor programs and parent support groups.

Providers who engaged in bidirectional communication practices were described as attentive, really “there for” women, and patient—a trait highly valued by women during a period when they felt “everything but patient” with themselves. Ultimately, women who engaged in bidirectional conversations with their providers felt that their concerns were heard and acknowledged, in turn fostering trust in and a desire to return to these providers for both breastfeeding concerns and routine care.

#### 2b. Lack of bidirectional communication.

Most women did not experience bidirectional communication with providers during their breastfeeding care, described by one woman as being spoken *at* instead of *with*. Many women shared that providers did not elicit or include their breastfeeding goals, knowledge, or concerns in conversations, as highlighted by one woman stating:

*There are no conversations, there are no significant—there is no integration between the knowledge that the obstetrician has and the disinformation that moms have. [35 years old, with previous breastfeeding experience]*

Most women felt under supported in their breastfeeding care, did not fully understand or receive all the information they considered necessary for their breastfeeding success, and/or perceived that their breastfeeding goals were not acknowledged or supported by their care team.

Providers who did not to engage in bidirectional communication with women instead used closed, checklist-style questions, assumed women’s breastfeeding goals, did not create opportunities in conversation for women to voice their concerns or need for clarification, and/or did not explain how women’s infant feeding goals were supported in the medical care plan.

To women, a lack of provider bidirectional communication in breastfeeding care fostered conversations that felt transactional and a sense that providers were uninvested in their breastfeeding experiences, both of which contributed to lower satisfaction and negative perceptions of care. Many women described providers during the delivery period as “[they] did their job and just left. [...] rushing to other people and not giving me the attention that I needed,” delivering “very quick,” “short” answers without clarification. Women felt that some providers considered breastfeeding conversations to be the role of the lactation specialists, with one woman sharing:

*Not all of them (providers) have that love, that grace to motivate. They can say, “That’s not my job, my job is to set up an IV, give medication, check on the baby, check on the mother and that’s it.” They can say, “That is not my job. That’s the job of a specialist,” you see? [33 years old with previous breastfeeding experience]*

A common, emotionally charged breakdown in communication for many women was a lack of bidirectional communication around infant formula supplementation. Many women felt that they received little to no explanation from providers on why formula was needed and/or how women’s breastfeeding goals would be supported during formula supplementation. Women reported significant disappointment in both them and their providers in these instances, as one woman shared:

*[I] thought, once I gave him formula, that was it, like I just had to give him formula from there on, so I was-I was really down about that. [25 years old, with previous breastfeeding experience]*

Some women expressed how improved communication on the indication for—and breastfeeding support during—formula supplementation could have alleviated significant maternal distress within the delivery and postpartum periods.

Lastly, many women reported that their care team provided pamphlets or packets as a replacement for verbal communication on breastfeeding altogether. These materials were most often provided close to discharge from the delivery hospitalization, a time when women felt too “overwhelmed” to read through papers. When asked how helpful these papers were, one woman shared:

*To be honest, I don’t think it was helpful at all because I was being discharged from the hospital. I had way too much. I had a brand-new baby. I had a bunch of other paperwork. I had a toddler at home. Paperwork wasn’t-wasn’t really something I was trying to be reading through in my spare time... When I was discharged, they said to me, “You know, inside this packet there’s a bunch of paperwork on breastfeeding and-and—you know... I mean, I just had a brand-new baby... I didn’t have time to be reading through papers. [26 years old, with previous breastfeeding experience]*

Moreover, a lack of bidirectional communication in breastfeeding care made women less trusting of or likely to return to such providers for breastfeeding or routine care, and was felt to needlessly enhance the stress, confusion, and overwhelm women experienced early in breastfeeding due to insufficient clarity around how women’s infant feeding goals were acknowledged and/or supported by the care team and broader medical plan.

## Discussion

Our study brings to light what constitutes effective communication in breastfeeding care and what provider practices both promote and hinder effective communication from the perspectives of Latina women in the U.S., a population of women facing breastfeeding inequities. We found that, for the Latina women in our study, there were two key elements of effective communication in breastfeeding care, including providers meeting women’s personal informational needs and engaging women in bidirectional conversations around breastfeeding. Effective communication experiences were found to promote positive perceptions of care and trust in women’s care team.

Aligned with our findings, women across nations and cultures have identified personalized and consistent communication around breastfeeding as key to their satisfaction with and perception of high-quality breastfeeding care [9–11], preferring communication that is tailored to their individual educational needs [22,58,59]. Prior evidence spanning chronological time, global geography, and sociodemographic groups supports women’s desire for more in-depth, realistic breastfeeding information from providers [12,27,37,39,44,46,60–63], frustration with inconsistent or incorrect messaging [9,10,18,22,35–37,39,41,44,47,63–71] and motivation around breastfeeding between providers [27,41,43,63,67], and perceptions that providers are rushed [10,22–24,41,43,44,48,69,71] or unable to lead breastfeeding conversations [12,44,46]. Our findings in Latina women also align with prior studies that found effective provider communication to be directly related to patient satisfaction with and confidence in their healthcare, improving care utilization and outcomes in breastfeeding and maternity care more broadly [1,4,11,22,35,72,73]. Moreover, there exists a critical gap in the ability of healthcare providers to meet women’s breastfeeding informational needs across the mother and infant care continuum in our current health system, a reality that is correlated to women having negative perceptions of their breastfeeding care [11,13,19,35,36,38–42] and maternity care more broadly [2,64,74].

Our findings extend existing literature in several ways. Schmied et al. (2011) described women’s perceptions and experiences of breastfeeding support in a meta-synthesis of 31 papers, four of which include U.S. Hispanic women [43–46], and none of which aimed to explore women’s experiences of provider communication nor Latina women’s perspectives of breastfeeding care, specifically. Of those studies that have since explored women’s perceptions of breastfeeding support broadly [13,19,35,36,38–42], those that included U.S. Hispanic women explored English-speaking individuals only [13,28], examined care in the delivery period exclusively [42], did not explore women’s care experiences with maternal and/or infant healthcare providers [48,49,69,75], or included very few Hispanic women in their sample (e.g., 3 of 98 women) [47]. Our study focused on the perspectives of Latina women, including both English-speaking and Spanish-speaking individuals, and captured their breastfeeding care experiences with a variety of providers across the maternal-newborn care continuum. Furthermore, our findings address a timely knowledge gap, as identified by two recent articles demonstrating the role of improved patient-provider communications as critical to improving hospital-based lactation support for Hispanic birthing individuals, and their trust in their maternity care more broadly [76,77].

Our finding that effective communication involves provider use of open-ended, exploratory questioning around breastfeeding is supported by Papautsky & Koenig’s earlier work observing lactation educators during delivery hospitalization. They demonstrated that women’s return to work and breastfeeding support systems were discussed in 75 and 80 percent of lactation education sessions, respectively, and that none of the observed sessions included an upfront inquiry of women’s breastfeeding goals [42]. Also aligned with our findings, earlier evidence supports that insufficient provider education and/or anticipatory guidance on breastfeeding challenges fosters women’s feelings of provider dishonesty and personal failure, shame, or guilt around infant feeding [19,28,35,58]. Our study adds to the growing body of literature that underscores the importance of meeting women’s breastfeeding informational needs across the maternal and infant care continuum to build and maintain women’s emotional wellbeing and trust in their care.

Latina women in our study also identified bidirectional communication as a key to effective communication in breastfeeding care. Bidirectional communication is more broadly known to augment individuals’ comprehension of health information and help elicit individual’s concerns to negotiate a mutual agenda for conversation between patient and provider, promoting person-centered care [78]. Yet, such bidirectional communication practices are not widely implemented in U.S. breastfeeding care. While observing lactation educators during delivery hospitalization, Papautsky & Koenig found that educators contributed nearly 85% of the average total words per session and asked an average of 12 questions (SD 5.1), compared with mothers who asked an average of 2 questions (SD 2.4) [42]. Such one-sided dynamics of communication in breastfeeding care lend support to women’s reported desire for more balanced, bidirectional conversations on breastfeeding instead of didactic, “one-way” conversations with their providers [10,11].

Schmied et al. (2011) found that women reported positive breastfeeding care experiences with providers who had an *authentic presence* (i.e., responsive, affirming, empathic) and a *facilitative* communication style (i.e., learner-centered approach that provides realistic, accurate, sufficiently detailed information) [11]. Similar to Latina women’s preference for bidirectional conversations in our study, facilitative communication was defined by Schmied et al. as enabling women to draw on both information and personal experience to build confidence and independent decision-making around breastfeeding. Also aligned with our findings, Schmied et al. identified negative experiences of care reported from women who received conflicting breastfeeding information in a didactic, pressured, or rushed manner (defined as *reductionist* and *disconnected* approaches) [11,79].

Several women within our sample reported ineffective communication due to an absence of bidirectional communication around infant formula supplementation, a major risk factor for the premature termination of breastfeeding [80,81], especially among Hispanic communities who have been observed to give “Las Dos Cosas” (i.e., feeding both breast milk and formula) [30,82–84]. Women perceived formula as given to their infant without their consent or without clear explanation as to the clinical indication, as seen in prior studies of other populations [36,47,65,85]. Moreover, innovative strategies to support more effective communication on formula supplementation are required to improve women’s experiences of their breastfeeding care and potentially prevent early cessation of breastfeeding due to misinformation and misunderstanding between patients and providers.

Spanish-speaking individuals, specifically, have identified bidirectional communication as important to both their breastfeeding and maternity care more broadly [86,87]. When there is a lack of bidirectional communication, opportunities arise for provider assumption(s) to influence breastfeeding information, education, and hands-on care received by patients, an experience reported by women in our study and others [65]. Such assumptions are likely to contribute to known racial and ethnic disparities in breastfeeding initiation and duration rates among U.S. women [29,88]. Strategies to improve the implementation of bidirectional communication practices between providers and women may mitigate the role of bias in breastfeeding care and improve breastfeeding outcomes for U.S. women [76,89].

Latina women in our study identified several systems level factors that negatively influence providers’ ability to practice effective communication practices. This included short clinical visits, high provider workload, varied levels of breastfeeding knowledge across providers, and lack of access to culturally and linguistically concordant providers or appropriate access to written and verbal Spanish translation. Lack of provider time has been recognized by both women [44,48,69] and providers [10,22–24,43] as a barrier to effective communication in breastfeeding care. Strong evidence supports that women experience rushed communication during their breastfeeding care across provider types (i.e., nursing, physicians, midwives, and lactation consultants) and settings (i.e., prenatal clinic, hospital delivery, postpartum clinic), underscoring this as a systems level issue [22–24,43,44,48,69,71]. Women consistently report frustration with inconsistent provider motivation to support breastfeeding [43,48,63,67]. Insufficient provider time and/or breastfeeding expertise to meet women’s breastfeeding informational needs may in part explain differences in communication between providers, as reported by providers themselves [12,22–27]. Thus, health systems should implement strategies to address these systems level factors to promote more effective provider communication. As suggested by our findings, such efforts could improve patient satisfaction with and trust in the care they receive, as well as patient engagement with care.

It is well documented that Hispanic women face barriers to effective communication due to a lack of provider cultural and linguistic diversity within U.S. health care systems [90,91]. Latina women in our study and others have felt that being cared for by culturally and/or linguistically concordant providers enhanced their communication experiences and that adequate translation services are needed to improve breastfeeding care for Hispanic women [76,77,92]. Identifying and addressing systems level factors to more effectively communicate with women has potential to meaningfully impact patient satisfaction, trust, and health outcomes, as women in our study reported increased provider trust, willingness to voice their questions or concerns, and ultimately return to providers for their care after experiencing effective communication in their breastfeeding care. One strategy for improving racial, ethnic, and linguistic concordance in breastfeeding care includes the integration of bilingual breastfeeding peer counselors and certified lactation counselors from Hispanic communities into healthcare teams [73,93–96]. Breastfeeding peer counseling programs, such as the Breastfeeding Heritage and Pride program led by the Hispanic Health Council, have been found to promote effective communication and improve breastfeeding outcomes for Latina women [73]. Additionally, the integration of doulas into breastfeeding care across the maternal and infant care continuum could be an effective and scalable strategy [97–99], though future studies are needed to examine the impact of doula care on breastfeeding care experiences and breastfeeding outcomes among Hispanic women [99].

We identified opportunities for improved communication across all provider types and care timepoints (i.e., pregnancy, delivery, postpartum). Our findings build upon prior literature demonstrating women’s preference for breastfeeding conversations to be initiated early in pregnancy and repeated across the maternal and infant care continuum [35,46,100], seen to enhance women’s satisfaction with both breastfeeding care and outcomes [26,35,38,63,65,69]. The prenatal and postpartum periods have been identified as critical gaps in breastfeeding care by women, with breastfeeding care largely concentrated during women’s delivery hospitalization [26,39,65]. Frequent interruptions for medical care, distractions from visitors, and maternal fatigue or overwhelm likely hinder effective communication around breastfeeding during the delivery period [42,58]. Thus, effective communication practices should be used by providers across the maternal and infant care continuum, as highlighted within our *“Framework for Effective Communication in Breastfeeding Care.”*

### Implications for clinical practice and research

Our findings support the need for improved care delivery at the individual and systems levels to achieve more equitable, person-centered breastfeeding care for U.S. families, promote patient trust in and engagement with the health system, and improve breastfeeding outcomes. Future studies should build upon our findings by working together with Latina women to co-design strategies to improve effective communication in breastfeeding care, including efforts to address systems level barriers to effective communication and using our *Framework for Effective Communication in Breastfeeding Care* to guide provider training on effective communication practices with women in the context of their breastfeeding care and beyond [96].

### Limitations and strengths

We acknowledge several of our study’s limitations. First, all women in our study had a prenatal intention to breastfeed and thus their perceptions of breastfeeding care are likely to differ from women who do not intend to breastfeed. Nonetheless, most U.S. families initiate breastfeeding [101], supporting the potential for our findings to be widely transferable to the care of U.S. families. Second, most women in our study (20 of 21 women) delivered at a Baby-Friendly Hospital, a designation granted to healthcare facilities who meet certain best practices standards for breastfeeding care during birth hospitalization [102]. Our findings may differ from the experiences of mothers who deliver at hospitals without this designation. Our study also had notable strengths, including our collaboration with community partners and a community research fellow with lived breastfeeding experience, our exploration of women’s experience spanning the maternal-infant care continuum, and our diverse sample of English and Spanish-speaking Latina women born within and outside of the U.S.

## Conclusion

For the Latina women in our study, effective communication in breastfeeding care was constituted of personalized, bidirectional breastfeeding conversations that met women’s individual informational needs, provided culturally tailored, consistent information, and were delivered across the maternal and infant care continuum. Our study’s findings and framework add to limited existing knowledge on Latina women’s communication experiences during breastfeeding care and could help inform the development of person-centered outcome measures and/or guide implementation strategies to promote more effective communication practices among providers caring for breastfeeding women. The latter efforts are needed to achieve high-quality, person-centered breastfeeding care and equitable breastfeeding outcomes in the U.S. and beyond.

## Supporting information

S1 Table Interview questions and example probes exploring Latina women’s experiences of breastfeeding care. This table provides interview questions and example probes used to interview of U.S. Latina women and explore their breastfeeding care experiences across the pregnancy, delivery, and postpartum period.
(DOCX)

## Abbreviations

CARE: Community Alliance for Research and Engagement
COREQ: Consolidated criteria for reporting qualitative studies
IRB: Institutional Review Board
SNAP: Supplemental Nutrition Assistance Program
U.S.: United States
WHO: World Health Organization
WIC: Women, Infants and Children
