# Supplementary material for: What is effective communication in breastfeeding care? Perspectives from Latina women
Source: PLoS One. 2025 Jun 26;20(6):e0325592. doi: 10.1371/journal.pone.0325592 (PMC12200710; doi:10.1371/journal.pone.0325592)
Supplement: S1 Table — This table provides interview questions and example probes used to interview of U.S. Latina women and explore their breastfeeding care experiences across the pregnancy, delivery, and postpartum period. (DOCX) [file pone.0325592.s001.docx]

**Supplementary Table. Interview Questions and Example Probes Exploring Latina Women’s Experiences of Breastfeeding Care**

| **Interview Questions** | **Example Probes** |
| --- | --- |
| **Opening questions and probes** | |
| 1. What are some of the reasons you wanted to breastfeed your baby? | - What was the most important reason? |
| 1. When you were pregnant, how long did you plan on breastfeeding? | - How did you make this decision? |
| 1. Were you able to meet your breastfeeding goal? Why/Why not? | - In what ways did breastfeeding go/not go the way you wanted it to? - What has gone well? - What were the challenges? |
| **Key questions and probes on women’s experiences of breastfeeding care** | |
| 1. During your prenatal visits, did you talk with health care providers about how to feed your baby?   [IF YES] Can you tell me about your experiences talking with health care providers about infant feeding during your prenatal visits?  [IF NO] What would you have liked to talk to your provider about? | [IF YES, PROBE]   - Who did you talk with about infant feeding? - What did they talk with you about? - How did you feel about how these conversations went?   [IF NO, PROBE]   - Who would you have liked to have spoken to? - What would you have wanted to go differently? |
| 1. Can you tell me about your experiences with breastfeeding support immediately after your baby was born? | [FOR QUESTIONS 5 – 8, PROBE]   - Who provided support? - What support did they provide? - What did you like? - What did you not like? - How helpful was this support? - How was the communication? What did you like about how the health care provider(s) spoke with you? What did you not like? |
| 1. What kind of breastfeeding support did you get from healthcare providers from the time you arrived at the maternity ward until you left, if any? |  |
| 1. During your baby’s visits to the doctor, what kind of infant feeding support did you get from health care providers, if any? |  |
| 1. During your health care visits after your baby was born, what kind of infant feeding support did you get from health care providers, if any? |  |
| **Closing questions** | |
| 1. Overall, how satisfied were you with the breastfeeding support you got from health care providers? | - Why? - What were you most satisfied with? - What were you least satisfied with? |
| 1. How did the breastfeeding support from health care providers affect your breastfeeding journey, if at all? | - How did the breastfeeding support affect your breastfeeding journey in a positive way, if at all? - How did the breastfeeding support affect your breastfeeding journey in a negative way, if at all? - How did the breastfeeding support affect your ability to meet your breastfeeding goal, if at all? |
| 1. In your opinion, what would make the breastfeeding support from health care providers better for Latina moms? | - What would make the breastfeeding support better during pregnancy? during labor and delivery? on the maternity floor? after your baby was born? - What would be the most important change? Why? |
| 1. Is there anything else you would like to add before we end our conversation? |  |
